# Short strong hydrogen bonds in proteins: a case study of rhamnogalacturonan acetylesterase

**DOI:** 10.1107/S0907444908017083

**Published:** 2008-07-17

**Authors:** Annette Langkilde, Søren M. Kristensen, Leila Lo Leggio, Anne Mølgaard, Jan H. Jensen, Andrew R. Houk, Jens-Christian Navarro Poulsen, Sakari Kauppinen, Sine Larsen

**Affiliations:** a Department of Chemistry, University of Copenhagen, Universitetsparken 5, DK-2100 Copenhagen, Denmark; b Department of Chemistry, University of Iowa, IA 52242, USA; c Novozymes, Novo Allé, DK-2880 Bagsvaerd, Denmark; d European Synchrotron Radiation Facility (ESRF), BP 220, F-38043 Grenoble CEDEX, France

**Keywords:** short hydrogen bonds, low-field NMR signals, rhamnogalacturonan acetylesterase

## Abstract

An extremely low-field signal (at approximately 18 p.p.m.) in the ^1^H NMR spectrum of rhamnogalacturonan acetylesterase (RGAE) shows the presence of a short strong hydrogen bond in the structure. This signal was also present in the mutant RGAE D192N, in which Asp192, which is part of the catalytic triad, has been replaced with Asn. A careful analysis of wild-type RGAE and RGAE D192N was conducted with the purpose of identifying possible candidates for the short hydrogen bond with the 18 p.p.m. deshielded proton. Theor­etical calculations of chemical shift values were used in the interpretation of the experimental ^1^H NMR spectra. The crystal structure of RGAE D192N was determined to 1.33 Å resolution and refined to an *R* value of 11.6% for all data. The structure is virtually identical to the high-resolution (1.12 Å) structure of the wild-type enzyme except for the interactions involving the mutation and a disordered loop. Searches of the Cambridge Structural Database were conducted to obtain information on the donor–acceptor distances of different types of hydrogen bonds. The short hydrogen-bond inter­actions found in RGAE have equivalents in small-molecule structures. An examination of the short hydrogen bonds in RGAE, the calculated p*K*_a_ values and solvent-accessibilities identified a buried carboxylic acid carboxylate hydrogen bond between Asp75 and Asp87 as the likely origin of the 18 p.p.m. signal. Similar hydrogen-bond interactions between two Asp or Glu carboxy groups were found in 16% of a homology-reduced set of high-quality structures extracted from the PDB. The shortest hydrogen bonds in RGAE are all located close to the active site and short interactions between Ser and Thr side-chain OH groups and backbone carbonyl O atoms seem to play an important role in the stability of the protein structure. These results illustrate the significance of short strong hydrogen bonds in proteins.

## Abstract 2

The short hydrogen bonds in rhamnogalacturonan acetylesterase have been investigated by structure determination of an active-site mutant, ^1^H NMR spectra and computational methods. Comparisons are made to database statistics. A very short carboxylic acid carboxylate hydrogen bond, buried in the protein, could explain the low-field (18 p.p.m.) ^1^H NMR signal.

## 1. Introduction

Hydrogen bonds play a pivotal role in the structure and function of proteins. Protein secondary structure is shaped by hydrogen bonds between atoms of the polypeptide backbone, and hydrogen bonds between protein side chains and sub­strates are fundamental for the catalytic function and specificity of enzymes (Gutteridge & Thornton, 2005 ▶). The strength of the hydrogen bond, that is the energy associated with its formation, shows great variations from around 100 kJ mol^−1^ for the strong very short O—H⋯O hydrogen bonds, with O⋯O distances of 2.5 Å or less, which have been shown to have covalent character (Emsley *et al.*, 1990 ▶; Flensburg *et al.*, 1995 ▶; Madsen *et al.*, 1998 ▶) to much weaker C—H⋯O interactions of a few kJ mol^−1^ (Desiraju & Steiner, 1999 ▶; Gu *et al.*, 1999 ▶). Spectroscopic and thermodynamic measurements as well as theoretical calculations (Olovsson & Jönsson, 1976 ▶; Jeffrey, 1997 ▶) have all shown that the distance between the hydrogen-bond donor and acceptor is a good indicator of the strength of a given hydrogen bond, *e.g.* a shorter distance between equivalent donor and acceptor atoms reflects a stronger hydrogen-bonding interaction.

Among the strongest are the very short strong O—H⋯O hydrogen bonds formed between carboxylic acid and carboxylate groups. In many textbooks Asp and Glu are presented as charged residues, in accordance with the p*K*_a_ values of free aspartic and glutamic acids of 3.9 and 4.2, respectively. This would imply that they are deprotonated at normal physiological pH, *i.e.* they exist as carboxylates. However, the local chemical environment in a protein can change the microscopic p*K*_a_ value of a carboxylic acid group significantly, keeping the carboxylic acid residues protonated at higher pH (Sawyer & James, 1982 ▶). An experimental p*K*_a_ value as high as 9.9 has been reported for an Asp residue in the reduced form of human thioredoxin (Qin *et al.*, 1996 ▶). It is noteworthy that Asp and Glu are often not considered as hydrogen-bond donors in the programs that are employed in the analysis of protein structures for hydrogen-bond inter­actions, for example the widely used *HBPlus* (McDonald & Thornton, 1994 ▶). Therefore, hydrogen bonds between carboxylic acid and carboxylate groups may be overlooked, as happened in an otherwise carefully conducted analysis of hydrogen bonds in proteins (Rajagopal & Vishveshwara, 2005 ▶).

The presence of short strong hydrogen bonds can also be detected in IR and NMR spectra, of which the latter are more suited for the study of proteins. The formation of short strong hydrogen bonds with partially covalent character causes a deshielding of the proton involved, giving rise to ^1^H NMR chemical shifts above 18 p.p.m. Such low-field proton signals and their relation to low-barrier hydrogen bonds (LBHB) considered to be important for catalysis have been extensively studied (Garcia-Viloca *et al.*, 1998 ▶; Cleland *et al.*, 1998 ▶; Del Bene *et al.*, 2000 ▶; Arnold & Oldfield, 2000 ▶). Experimental and theoretical studies have shown unequivocally that proton chemical shifts higher than or around 18 p.p.m. correspond to strong short hydrogen bonds, although the role of LBHB in catalysis is still disputed (Schutz & Warshel, 2004 ▶).

The serine proteases with a catalytic Asp-His-Ser triad containing a short Asp–His hydrogen bond were among the systems investigated (Cleland *et al.*, 1998 ▶). A virtually identical catalytic Asp-His-Ser triad was also found in rhamno­galacturonan acetylesterase (RGAE) and the esterase catalysis is assumed to follow a similar mechanism (Mølgaard *et al.*, 2000 ▶). The structure of RGAE from *Aspergillus aculeatus* is known in two different crystal systems: a trigonal form and an orthorhombic form to very high (1.12 Å) resolution (Mølgaard & Larsen, 2002 ▶, 2004 ▶). Analysis of the structure of RGAE and comparison with structurally related enzymes led to the initial characterization of the SGNH-hydrolase family (Mølgaard *et al.*, 2000 ▶), which is now defined as a superfamily in SCOP (Murzin *et al.*, 1995 ▶). Despite very low sequence identity, the members of this family have four characteristic blocks of conserved residues. The study of wild-type RGAE also included ^1^H NMR measurements, which revealed signals from two deshielded protons at 18.2 and 14 p.p.m. (Mølgaard, 2000 ▶). As a ^1^H NMR signal above 18 p.p.m. had previously been observed in the serine protease α-chymotrypsin (Cassidy *et al.*, 1997 ▶), our first hypothesis was that the 18 p.p.m. signal had its origin in the possible low-barrier hydrogen bond between His195 and Asp192 in the active site, which has an N—O distance of 2.63 Å. In order to examine this hypothesis, a variant of RGAE was prepared in which the catalytic negatively charged Asp was replaced with an Asn. However, the ^1^H NMR spectra of this D192N variant of RGAE were not as easily interpretable as expected, as it also showed an 18 p.p.m. signal like wild-type RGAE. This prompted a more thorough investigation of all the short hydrogen bonds in the enzyme. The results presented here comprise the determination of the crystal structure of the D192N variant, measurements of the ^1^H NMR spectra of the wild-type and D192N variant of RGAE as a function of pH, complemented by theoretical calculations of proton chemical shifts and p*K*_a_ values for specific residues. We have used these results in combination with a careful and exhaustive analysis of the potential short hydrogen bonds in RGAE, based on bond-distance analysis from atomic resolution structural data in the Cambridge Structural Database, to provide an interpretation of the ^1^H NMR spectra. A hydrogen bond between two buried Asp residues was shown to match the experimental data. A search of a representative subset of structures in the Protein Data Bank (PDB; Berman *et al.*, 2000 ▶) revealed that this type of interaction is not uncommon in proteins.

## 2. Methods and materials

### 2.1. Expression and purification

The D192N mutant (numbering corresponding to that of Mølgaard *et al.*, 2000 ▶) of *A. aculeatus* RGAE was generated by standard molecular-biology methods and cloned into the pHD464 plasmid. The resulting construct was transformed into *A. oryzae* (Christensen *et al.*, 1988 ▶) for overexpression. RGAE D192N was purified from the *A. oryzae* culture supernatant by a procedure similar to that used for wild-type RGAE (Kauppinen *et al.*, 1995 ▶). As further purification of the variant was necessary, RGAE D192N was subjected to an additional size-exclusion chromatography step using a Superdex 75 (300 ml) column run with 20 m*M* MES, 0.1 *M* NaCl pH 6.0. Selected fractions were pooled and dialyzed against 20 m*M* MES pH 6.0 and concentrated to a protein concentration of about 30 mg ml^−1^ (BCA-assay) by means of a Centriprep 10 (Amicon). Judged from the SDS–PAGE, RGAE D192N was more than 95% pure.

### 2.2. Crystallization of RGAE D192N

Crystallization trials were unsuccessful using conditions similar to those used to obtain crystals of wild-type RGAE, possibly owing to small variations in glycosylation, as different expression runs have previously been shown to yield heterogenous glycosylation of the two N-glycosylation sites (Mølgaard *et al.*, 1998 ▶). Needle-shaped crystals were obtained with Hampton Crystal Screen I (condition No. 43) and optimization of the conditions resulted in crystals suitable for diffraction experiments. The crystal used for structure determination was obtained by the vapour-diffusion method using hanging drops at room temperature with a solution of 25% PEG 1500 in a 0.1 *M* sodium acetate buffer pH 3.0 as the precipitant and reservoir. The setup was made with drops composed of 4 µl protein solution and 2 µl reservoir solution equilibrated against 1 ml reservoir solution.

### 2.3. X-ray data collection and processing

A set of X-ray diffraction data was collected at Elettra using a wavelength of 1.00 Å and a MAR165 CCD detector. PEG 400 was added to a small amount of reservoir solution, resulting in a 15%(*v*/*v*) solution, which was used as a cryoprotectant. The crystal was cooled to 100 K during data collection.

Data were collected to a resolution limit of 1.33 Å. Analysis of the data showed that the crystal belonged to space group *P*2_1_2_1_2_1_, with unit-cell parameters *a* = 48.61, *b* = 67.61, *c* = 73.27 Å. With one molecule in the asymmetric unit, this corresponds to a Matthews coefficient of 2.45 Å^3^ Da^−1^ and a solvent content of ∼50%. The space group is the same as for the wild-type enzyme (*P*2_1_2_1_2_1_; *a* = 52.14, *b* = 56.87, *c* = 71.89 Å; Mølgaard *et al.*, 2000 ▶), but the unit-cell parameters are distinctly different. Indexing, integration and merging of data images were carried out using *DENZO* and *SCALEPACK* (Otwinowski & Minor, 1997 ▶). Statistics of the data collection and analysis are listed in Table 1 ▶.

### 2.4. Structure solution and refinement

The structure was solved by molecular replacement with the program *EPMR* (Kissinger *et al.*, 2001 ▶) using reflections in the resolution range 15–4 Å and the high-resolution structure of wild-type RGAE (PDB code 1k7c) as the search model. The structure was refined using the conjugate-gradient algorithm (CGLS) in *SHELXL*97 (Sheldrick, 2008 ▶) with statistical weights from the data collection. Initial refinement included positional parameters, isotropic displacement parameters, a preliminary water structure (156 water molecules), two *N*-­acetyl-d-glucosamine moieties and an acetate ion bound in the active site (*R*_work_ = 20.78% and *R*_free_ = 23.10% for all data with no *F*_obs_ cutoff). Introducing anisotropic displacement parameters for all non-H atoms reduced *R*_work_ and *R*_free_ to 15.87% and 19.50%, respectively. Five residues (Gly77–Thr81) were poorly defined in the electron-density maps. Therefore, Gly77 was modelled in two different conformations with a total occupancy of 1, and Ser78 and Thr81 were modelled with an occupancy of 0.5, while Leu79 and Ser80 were not included in the model. Additionally, 11 residues were modelled with two different side-chain conformations and one residue (Ser32) with two different conformations of the entire residue. Refinement of the alternative conformations and introduction of additional water molecules yielded *R*_work_ and *R*_free_ values of 12.52% and 16.80%, respectively.

Introduction of H atoms reduced the *R* values by 1%; riding H atoms were not added to hydroxyl and carboxyl groups, nor was His protonization introduced in the model. In the end, refinement against all data (work and free set) was performed and a final round of full-matrix least-squares refinement of the positional parameters was carried out to obtain the estimated standard deviation on the coordinates and selected inter­atomic distances (using keywords L.S. 1, DAMP 0 0, BLOC 1). Statistics for the final model are listed in Table 1 ▶. Excluding disordered and riding atoms, the radial positional e.s.d.s calculated in *SHELXL* are in the range 0.02–0.17 Å for the protein atoms in RGAE D192N. The e.s.d.s in specific directions are the radial positional e.s.d. divided by 3^1/2^. However, the e.s.d.s of the distances in Table 4 were calculated specifically using the HTAB keyword in the full least-squares refinement. In the calculations of the e.s.d.s on the distances, a 0.1% uncertainty on the unit-cell dimensions was employed (the default parameter from *SHELXPRO*). A similar round of least squares-refinement was performed for the wild-type RGAE to obtain calculated e.s.d.s for the interatomic distances.

Global estimates of the accuracy of the atomic positions were obtained from *SFCHECK* (Vaguine *et al.*, 1999 ▶) in the form of an estimated maximal error (EME; Cruickshank, 1949 ▶) and a diffraction precision index (DPI; Cruickshank, 1999 ▶). For the RGAE D192N structure EME = 0.034 Å and DPI = 0.041 Å; the corresponding values for the wild-type structure were 0.027 and 0.034 Å, respectively. An analysis of bond-length directionality using *WHAT IF* (Vriend, 1990 ▶) indicated no significant systematic deviations. The global and local error estimates are in agreement and for the short distances of the hydrogen bonds investigated (Table 4) the calculated e.s.d.s are in the range 0.01–0.04 Å. For significant differences in distances the uncertainty should be multiplied by at least a factor of three (Weber *et al.*, 2007 ▶), so only structural differences of the order of 0.1 Å in the well ordered regions should be considered.

Hydrogen bonds in the structure of the mutant as well as in wild-type RGAE (PDB code 1k7c) were calculated using the program *HBPLUS* (McDonald & Thornton, 1994 ▶) using the option to search for neighbouring atoms rather than strict hydrogen bonds, as Asp and Glu are not included as potential hydrogen-bond donors. Contacts within a residue, with the nearest sequential neighbour and with water molecules were excluded from further analysis. The relative solvent-accessible surface for the residues was calculated using *NACCESS* (Hubbard & Thornton, 1993 ▶) with a default probe size of 1.4 Å. All figures of the structure or part of the structure were produced using *PyMOL* (DeLano, 2002 ▶).

### 2.5. Hydrogen-bond geometry extracted from the databases

The Cambridge Structural Database (Allen, 2002 ▶) was searched to obtain information on the donor–acceptor distances of different types of possible short strong hydrogen bonds that could be expected in a protein structure. All searches were performed with the *ConQuest* program (Bruno *et al.*, 2002 ▶) using filters so that only organic structures with *R* < 5% without disorder and errors were included. Polymeric structures and structures based on powder diffraction experiments were excluded. The results of the searches were analysed using *Vista* (CCDC, 1994 ▶). The search models were based on different functional groups (see Tables 2 ▶ and 3 ▶) mimicking the hydrogen bonds that can be formed in a protein. The sum of the van der Waals radii (O, 1.52 Å; N, 1.55 Å) and a *D*—H⋯*A* angle larger than 120° were used as cutoffs.

As RGAE does not contain any free S—H groups (only two S—S bridges), the search was focused on interactions between functional groups containing O and N. We combined systems in cases where position (*i.e.* main-chain or side-chain amide) or different protonization states did not lead to significant differences in the average hydrogen-bond lengths.

A reduced set from the Protein Data Bank (Berman *et al.*, 2000 ▶) was examined for putative hydrogen bonds between Asp and Glu residues. The reduced set comprised 3556 protein chains from a CulledPDB set from the *PISCES* server (Wang & Dunbrack, 2003 ▶) obtained in November 2006. The protein chains in the set had a sequence identity below 30% and were from structures determined from diffraction data to 2 Å resolution or better and with *R* < 25%. The search was carried out by Python scripts using the *Bio.PDB* package (Hamelryck & Manderick, 2003 ▶).

Disordered residues were excluded and by using a distance cutoff of 2.9 Å we assumed that most O⋯O contacts at metal sites were excluded (Flocco & Mowbray, 1995 ▶). The DPI was calculated for the protein chains in the set whenever the PDB header included the necessary information (approximately 75% of the structures). The DPI values were in the range 0.01–0.25 Å, confirming the quality of the structures of the set. The one exception with a DPI of 0.35 Å did not have close contacts between carboxylate groups. For protein chains with very short contacts between carboxylic acid residues (O⋯O < 2.6 Å) the Catalytic Site Atlas (Porter *et al.*, 2004 ▶) was used to find information on annotated or putative catalytic residues.

### 2.6. NMR experiments

One-dimensional ^1^H NMR spectra were recorded for wild-type RGAE and RGAE D192N. A Varian UNITY INOVA 500 MHz spectrometer was used to measure the spectra at 270 K. The spectra were measured with a total recording time of 25 min for the wild type and 1 h 8 min for the RGAE D192N. The spectra were indirectly referenced to TMS by assigning the water resonance a chemical shift of 5.11 p.p.m.

^1^H NMR experiments were performed on wild-type RGAE in a solution with a composition identical to the conditions at the beginning of the wild-type RGAE crystallization experiment [40 mg ml^−1^ RGAE, 1.4 *M* (NH_4_)_2_SO_4_ and 0.1 *M* sodium acetate buffer pH 5.0]. To examine the effect of the high SO_4_^2−^ concentration, spectra were also recorded under SO_4_^2−^-free conditions both in the presence of acetate buffer and in pure water.

The effect of pH was examined by performing titration series of wild-type RGAE and RGAE D192N. For wild-type RGAE, the pH was varied from pH 3.67 to 11.2 in eight steps. Below pH 3.67 the enzyme precipitated and above pH 11.2 it denatured. Experiments were performed in 0.1 *M* acetate buffer and without buffer. There was no noticeable difference in the NMR spectra corresponding to these latter two conditions. For RGAE D192N, ^1^H NMR spectra were recorded at seven different pH values in the range 6.0–10.1.

### 2.7. Calculation of p*K*_a_ values

The p*K*_a_ values for side chains in RGAE and D192N RGAE were predicted using the *PROPKA* 1.00 web interface (http://propka.ki.ku.dk) based on the high-resolution X-ray structure of wild-type RGAE (PDB code 1k7c). The *PROPKA* method is based on a set of empirical rules relating various aspects of protein structure (desolvation, hydrogen bonding and interactions between charged residues) to the p*K*_a_ values of amino-acid residues in proteins. The method has been shown to give p*K*_a_ values within ±1 pH unit of experimentally determined p*K*_a_ values (Li *et al.*, 2005 ▶).

### 2.8. Calculation of proton chemical shifts

Small structural models of the environment of the short strong hydrogen bonds between Asp75 and Asp87, Asp192 and His195, and Glu70 and His169 were constructed based on the wild-type RGAE crystal structure (to which H atoms had been added using the *PDB*2*PQR* web interface; Dolinsky *et al.*, 2004 ▶). Since the primary goal of these calculations was to determine whether the chemical shifts of the protons involved in the hydrogen bonds were near 18 p.p.m., relatively small structural models were constructed for computational efficiency. The models include groups directly hydrogen bonded to the residues of interest.

For the Asp75–Asp87 hydrogen bond, only the positions of the COOH–OOC atoms were energy-minimized at the B3LYP/6-31G(d) level of theory. Similarly, only the positions of the COO–imidazole atoms were energy-minimized for the Asp192–His195 and Glu70–His169 hydrogen bonds (except the position of the C^δ2^ carbon, which was not energy-minimized in the latter case). Since Asp192–His195 is close to the protein surface, the energy minimization was performed in the presence of a continuum description of bulk solvation (Li & Jensen, 2004 ▶).

Prediction of ^1^H NMR chemical shifts presents a challenge to theory owing to the very high level of theory necessary for converged results and the effect of the molecular environment (*e.g.* solvent or protein). Chesnut (1996 ▶) has proposed a linear scaling technique to address these effects and Rablen *et al.* (1999 ▶) have obtained the necessary parameters for proton chemical shifts relative to TMS in nonpolar solvents (CDCl_3_ and CCl_4_), 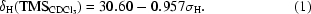
Here, σ_H_ is the isotropic chemical shielding calculated at the B3LYP/6-311++G(d,p)//B3LYP/6-31G+(d) level of theory, which in this study is approximated by B3LYP/6-311++G(d,p)//B3LYP/6-31G(d). The aqueous phase value of the chemical shielding of the proton is given by Porubcan *et al.* (1978 ▶), 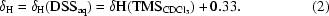
This correction is almost entirely owing to solvent effects, since DSS and TMS have very similar chemical shifts in the same solvent (Harris *et al.*, 2001 ▶). This approach was used previously by Molina & Jensen (2003 ▶) to successfully predict proton chemical shifts in the active sites of α-chymotrypsin and α-lytic protease. The chemical shielding calculations were performed with the *PQS* program on an eight-node QuantumStation, while the constrained geometry optimizations were performed with the *GAMESS* (Schmidt *et al.*, 1993 ▶) program.

## 3. Results

### 3.1. Structure of RGAE D192N

**Figure 1:**
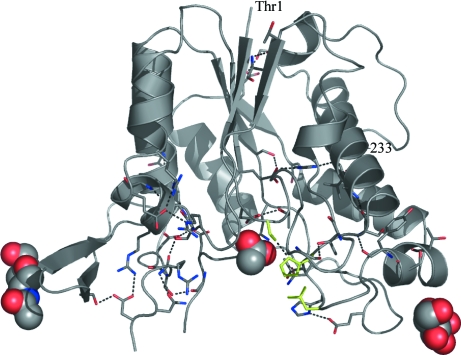
Overall structure of RGAE D192N excluding Thr79 and Ser80, which could not be located in the density maps. The terminal residues Thr1 and Leu233 are labelled. The three residues corresponding to the catalytic triad (Ser9-His195-Asn192) are coloured green and the GlcNAc moieties and acetate ion are illustrated by spheres. The short hydrogen bonds in Table 4 ▶ are shown as dashed lines.

**Figure 2:**
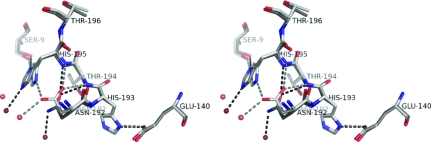
Stereo figure showing the active-site His195–Asp192/His195–Asn192 and His193–Glu140 interactions in wild-type RGAE and RGAE D192N in lighter and darker shades, respectively.

The structure of RGAE D192N, shown in Fig. 1 ▶, is highly similar to the structure of wild-type RGAE (r.m.s.d. of 0.43 Å for C^α^ atoms compared with PDB entry 1k7c) except for a disordered loop (Gly77–Thr81) and the active site, where significant changes can be observed in the hydrogen-bonding pattern on replacing the catalytic Asp with Asn (Fig. 2 ▶). The short Asp–His hydrogen bond observed in the wild type is not present and the corresponding distance between Asn192 and His195 is approximately 0.8 Å longer.

The conformation of His195 in RGAE D192N appears ambiguous; analysis of the hydrogen-bond pattern indicates that His195 is rotated relative to its conformation in wild-type RGAE. As in wild-type RGAE, the imidazole group of His195 is hydrogen bonded to Ser9 (2.73 Å), whereas a hydrogen bond to a water molecule (2.79 Å) has replaced the hydrogen bond to Asp192. The shortest distance (3.36 Å) between any two atoms of Asn192 and His195 is between His C^δ2^ and Asn O^δ1^.

**Figure 3:**
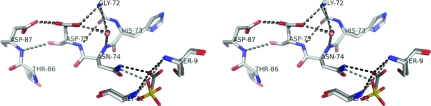
Stereo figure showing the Asp75–Asp87 area and oxyanion hole in wild-type RGAE and RGAE D192N in lighter and darker shades, respectively.

The high-resolution orthorhombic structure of the wild-type enzyme contains a sulfate ion in the active site, with one of its O atoms in the oxyanion hole forming hydrogen bonds to Ser9 N, Gly42 N and Asn74 N^δ2^. In RGAE D192N an acetate ion is bound in a similar position, with an O atom occupying the oxyanion hole almost in the same position as the O atom of the sulfate ion in wild-type RGAE (see Fig. 3 ▶).

One loop (Gly77–Thr81) was poorly defined in the electron density of RGAE D192N. It is evident from the crystal packing that this loop cannot be in the same conformation as observed in the structure of the wild type and the disorder is most likely to be a result of differences in crystal packing and not a consequence of the D192N mutation.

The glycosylation sites (Asn104 N^δ2^ and Asn182 N^δ2^) each have an *N*-acetyl-d-glucosamine (GlcNAc) moiety bound. The additional mannose residues observed in the orthorhombic structure of wild-type RGAE were not visible in the electron-density maps of RGAE D192N. These mannose residues take part in crystal contacts in the structure of the wild type; thus, differences in the degree of glycosylation could be a cause of the differences in the crystal packing of RGAE D192N and wild-type RGAE.

#### 3.1.1. Crystal packing

Wild-type RGAE has previously been crystallized in both an orthorhombic and a trigonal space group at pH 5 and pH 4.5, respectively (Mølgaard *et al.*, 1998 ▶). The two crystal forms were observed in the same drop at pH 4.7. From an analysis of the crystal packing of the two forms (Mølgaard & Larsen, 2004 ▶), it was concluded that one of the crystal contacts depends on the protonation state of several Glu residues on the surface of the protein (Glu202 and Glu206 from one molecule and Glu94 from a symmetry-related molecule). At higher pH values (above ∼4.7, corresponding to the crystallization conditions for the ortho­rhombic form) Glu202 and Glu206 form a very short intramolecular hydrogen bond (2.49 Å), implying that only one of the residues is deprotonated. At lower pH (below 4.7, as in the crystallization conditions for the trigonal form) these Glu residues are involved in different crystal con­tacts including intermolecular contacts between Glu202 and Glu94. An analysis of the packing of RGAE D192N revealed that contacts involving Glu residues 94, 202 and 206 are similar to those of the trigonal wild-type form. This agrees very well with the expected protonation state of the Glu residues (94, 202 and 206) as RGAE D192N was crystallized from a solution containg sodium acetate buffer pH 3.0. The other contacts between molecules in the RGAE D192N crystal structure were unique to this modification.

#### 3.1.2. Characterization by NMR

The ^1^H NMR spectra showed the characteristic appearance of a folded protein with fine structure in both the aliphatic, aromatic and amide regions (not shown). There was a distinct signal at 18.2 p.p.m. both in spectra with and without SO_4_^2−^. The spectra recorded in the crystallization solution were slightly broadened (not shown), presumably as a consequence of aggregation. The signal at 18.2 p.p.m. disappeared at temperatures above 310 K as a consequence of solvent saturation transfer when the water signal was presaturated (Grzesiek & Bax, 1993 ▶). This is consistent with previous findings in ^1^H NMR studies of chymotrypsin (Markley & Westler, 1996 ▶). At lower temperatures the intensity of the signal increases, which could be indicative of a decrease in the exchange rate.

**Figure 4:**
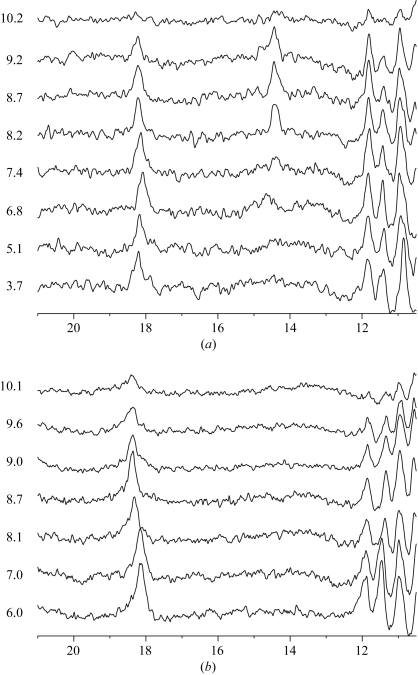
^1^H NMR spectra of wild-type RGAE (*a*) and RGAE D192N (*b*) at various pH values.

Spectra for wild-type RGAE were measured at eight different pH values ranging from pH 3.67 to 10.2 (Fig. 4*a* ▶). The 18.2 p.p.m. signal was present over the entire pH range. At pH 10.2 denaturation results in reduced signals in the whole spectrum. A second low-field signal at approximately 14 p.p.m. appeared at pH 7.4. Spectra for RGAE D192N were measured at seven different pH values between pH 6.0 and 10.1 and are shown in Fig. 4 ▶(*b*). A low-field signal at 18.2 p.p.m. was also present in these spectra as observed for the wild type. The activity measurements on the D192N sample ruled out deamidation of Asn192 as the source of the 18 p.p.m. signal. The signal observed around 14 p.p.m. at pH 7.4 and above in the spectra of the wild type could not be detected in the spectra of the mutant.

#### 3.1.3. Expected hydrogen-bond lengths in proteins based on the analysis of small-molecule structures

Tables 2 ▶ and 3 ▶ summarize the results of searches of the Cambridge Structural Database for hydrogen-bond lengths and angles in small molecules that mimic the hydrogen-bond interactions between different functional groups in proteins. We found it important that the averaged lengths and angles should have a statistically sound basis; therefore, we have only included bond lengths and angles that are averaged over at least five fragments. We also assume that hydrogen-bonded systems which can be found less than five times in the CSD searches are not very likely to be detected in proteins.

**Figure 5:**
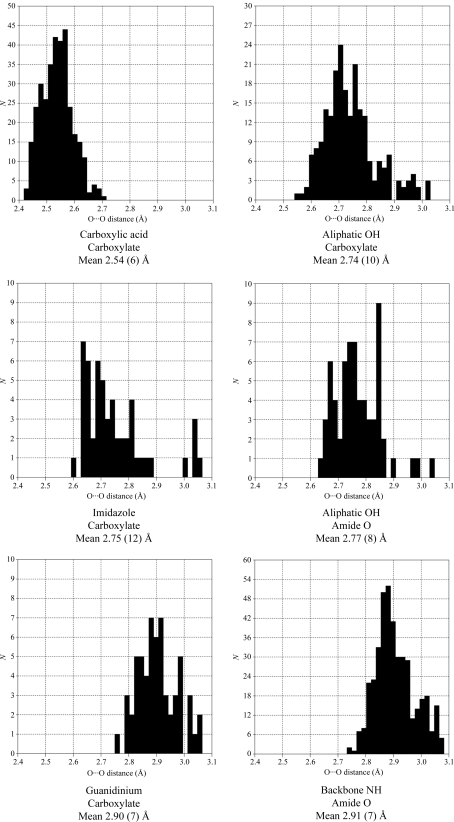
Histograms showing the distribution of distances in the Cambridge Structural Database for some of the short hydrogen-bond types present in RGAE.

The hydrogen-bond lengths and the statistical standard deviation in Tables 2 ▶ and 3 ▶ for each particicular type of interaction represent the average of distances that fall in a fairly broad range (0.3–0.5 Å), as illustrated by the histograms in Fig. 5 ▶. The distributions which include more than 100 fragments all show well defined maxima.

The average O⋯O distance of the different types of O—H⋯O hydrogen bonds listed in Table 2 ▶ ranges from 2.54 (6) to 2.82 (9) Å. The variation in O⋯O distances reflects the differences in p*K*_a_ values of the different donor/acceptor group, *e.g.* the O⋯O distance is shorter for a phenol carboxylate hydrogen bond than for alcohol carboxylate interactions. However, if one considers the standard uncertainty on the averaged hydrogen-bond lengths, these two interactions are barely distinguishable. All the search fragments contained selected H atoms and/or charges, which in principle enabled differentiation between the hydrogen bonds formed with the groups as either donor or acceptor, *e.g.* the interaction between carboxylic acid and alcohol groups. The length of the two types of hydrogen bonds [2.65 (5) and 2.81 (9) Å] is so similar that only in protein structures determined to atomic resolution is it possible to distinguish between the two types of interactions and thus be able to determine which of the two O atoms is the proton donor. The shortest O⋯O hydrogen bond is observed between a carboxylic acid and a carboxylate group. The distribution in donor–acceptor distances for this system (Fig. 5 ▶) is among the most narrow. An interesting result from this analysis is that the carboxylic acid–amide O hydrogen bond has a similar narrow distribution and only a slightly longer donor–acceptor distance of 2.60 (5) Å.

The donor–acceptor distances for the N—H⋯O hydrogen bonds listed in Table 3 ▶ are significantly longer than the O—H⋯O interactions and range from 2.75 (12) to 2.98 (4) Å. The analysis of the different types of hydrogen bonds is based on fewer fragments than was the case for the O⋯O hydrogen bonds; only two contain more than 100 examples, namely aliphatic NH^+^–carboxylate and amide NH–amide O. Fig. 5 ▶ shows the well defined distribution for the latter around the average 2.91 (7) Å. The shortest N—H⋯O hydrogen bond of 2.75 (12) Å is between imidazole and carboxylate groups, mimicking that found between the His and Asp residues in a catalytic triad. The other possible side chain–side chain hydrogen bonds that involve a carboxylate group mimicking Asp/Glu interactions with Lys and Arg are longer at 2.82 (8) and 2.90 (9) Å, respectively. The donor–acceptor dis­tances show the same gradual variation as described above for the O—H⋯O hydrogen bonds, reflecting the difference in p*K* values, *e.g.* a shorter hydrogen bond corresponds to a smaller difference in the p*K* values. The difference in N⋯O distance for hydrogen bonds involving identical functional groups with different protonation, *e.g.* imidazole–carboxylic acid [2.82 (8) Å] and imidazole–carboxylate [2.75 (12) Å], is so small that it can hardly be detected even in protein structures determined to the highest resolution. The two structures of RGAE were both determined to high resolution and the standard uncertainties on the distances were estimated to be in the range 0.01–0.04 Å from the least-squares refinement, which opens possibilities for comparison with the results obtained from the analysis of small-molecule structures. The short strong hydrogen bonds could be expected to have donor–acceptor distances between 2.5 and 2.6 Å (Jeffrey, 1997 ▶). To make sure that no possible short hydrogen bonds are excluded from our analysis, we have examined the wild-type RGAE and RGAE D192N structures for O⋯O distances less than 2.75 Å and for N⋯O distances less than 2.80 Å.

#### 3.1.4. Short hydrogen bonds in RGAE

Excluding residues that are poorly defined owing to disorder (multiple conformations), the distances between possible donor and acceptor atoms that are shorter that the cutoff distances in either wild-type RGAE, RGAE D192N or both are listed in Table 4 ▶ together with the calculated p*K*_a_ of the functional groups and solvent-accessibility. The positions of these hydrogen bonds in the RGAE D192N structure are illustrated in Fig. 1 ▶. It is noteworthy that all the hydrogen bonds identified as the shortest in the structure are located close to the active site. The hydrogen bonds in RGAE can all be recognized as one of the types of interactions listed in Table 2 ▶ and 3 ▶, with the general comment that the donor–acceptor distances observed in RGAE are shorter than the average distance derived from the analysis of small-molecule structures. The shortest O⋯O distances (below 2.5 Å) are observed between carboxylic acid and carboxylate groups. Another set of relatively short side-chain interactions are found between hydroxy groups (Ser, Thr, Tyr) and carboxylate groups (Asp, Glu), with O⋯O distances from 2.56 (2) Å. Although they should be considered as hydrogen bonds of medium strength, it is very likely that these interactions are important for the stability of the protein. Of similar importance are the hydrogen bonds between hydroxy groups and backbone or side-chain amide O atoms, which have O⋯O distances around 2.6 Å, which is much shorter than the average for this type of interaction in small molecules (Table 2 ▶). The calculated p*K*_a_ values of the residues involved in short hydrogen bonds are included in Table 4 ▶. The proton chemical shifts were calculated for selected hydrogen bonds that were possible candidates for the low-field chemical shift. The predicted values were 18.1 p.p.m. for His195 N^δ1^–Asp192 O^δ2^, 18.4 p.p.m. for His169 N^δ1^–Glu70 O^∊1^ and 18.5 p.p.m. for Asp75 O^δ2^–Asp87 O^δ2^.

#### 3.1.5. Carboxylic acid interactions in the PDB subset

The amino-acid composition of the analysed set with respect to Asp and Glu has been compared with UniProtKB/Swiss-Prot protein statistics (knowledge-base release 54.3; Bairoch *et al.*, 2005 ▶). Asp comprises 5.84% of the amino acids in the analysed set, which is slightly higher than the UniProtKB/Swiss-Prot statistic of 5.34%. The statistics for Glu did not differ (6.6% of the total amino acids).

With the cutoff at 2.9 Å, we found interactions between carboxylic acid side chains in 566 protein chains (16% of the total) distributed on 154 chains with Asp–Asp contacts, 226 with Glu–Glu contacts and and 311 with Asp–Glu contacts.

In 308 (9%) of the chains the O⋯O distances for Asp–Asp, Asp–Glu or Glu–Glu were shorter than 2.6 Å. These chains were investigated further for evidence of enzymatic activity and catalytic residues. It was possible to find annotated or putative catalytic residues for 142 of these structures using the Catalytic Site Atlas (CSA; Porter *et al.*, 2004 ▶). In 30 of the 142 chains (21%) one or more of the carboxylic acid residues involved in a short contact were annotated or putative catalytic residues. An additional 17 chains (12%) had the carboxylic residue within three residues of a putative catalytic residue in the sequence.

## 4. Discussion

### 4.1. Short hydrogen bonds in RGAE

One of the goals of the current study was to analyze the short hydrogen bonds in RGAE with the purpose of identifying the likely candidate for the deshielded proton that gives rise to the 18 p.p.m. signal in the ^1^H NMR spectra. The observation that almost all the short hydrogen bonds in RGAE listed in Table 4 ▶ are located in a region close to the active site (Fig. 1 ▶) shows the significance of these interactions.

Although the structure of RGAE is known to high resolution, it does not enable us to determine the exact protonization state of the side chains; thus, we have based our analysis of the different hydrogen bonds on the distance between the donor and acceptor atoms, the calculated p*K*_a_ values and the solvent accessibilities combined with results from small-molecule structures.

#### 4.1.1. The O—H⋯O hydrogen bonds

**Figure 6:**
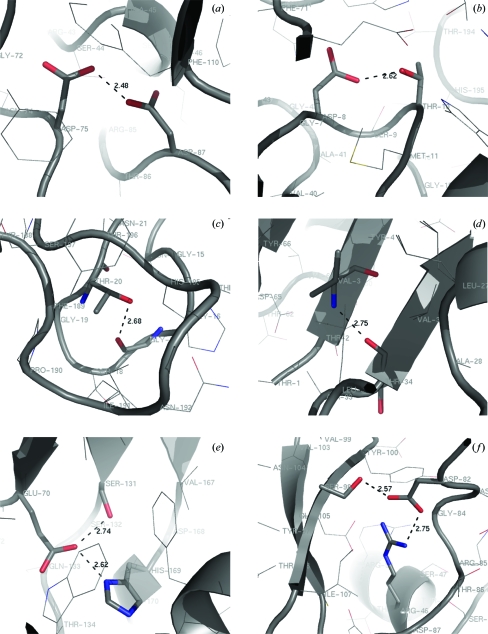
Examples of different types of short hydrogen bonds from the RGAE D192N structure. (*a*) Asp75–Asp87, (*b*) Thr10–Asp8, (*c*) Thr20–Gly17, (*d*) Val3–Thr34, (*e*) His169–Glu70 and Ser131–Glu70, (*f*) Arg46–Asp82 and Ser98–Asp82.

O—H⋯O hydrogen bonds with O⋯O distances shorter than 2.75 Å represent three different types of interactions. In accordance with the results from the analysis of small-molecule structures, the shortest is between the carboxy groups of Asp75 and Asp87 (Fig. 6 ▶ *a*), 2.47 (1) and 2.48 (2) Å in wild-type RGAE and RGAE D192N, respectively. The similar interaction between Glu202 and Glu206 observed in the orthorhombic structure of the wild type is not observed in the RGAE D192N structure, where the residues are involved in crystal contacts as described in §[Sec sec3.1.1]3.1.1. The two other types of short hydrogen bonds have side-chain OH groups as donor. Five hydrogen bonds with O⋯O distances in the range 2.56 (2)–2.75 (1) Å connect Ser/Thr side chains with the carboxylate groups from Asp/Glu residues in both RGAE structures. The O⋯O distances in the two structures are virtually identical, with one distance almost as short as the O⋯O distance in the carboxylic acid carboxylate hydrogen bond and significantly below the average distance of the similar interaction in small-molecule structures at 2.74 (10) Å. Two of these interactions are completely buried in the protein [Thr10–Asp8 (Fig. 6 ▶ *b*) and Ser131–Glu70]. Although the O⋯O distances could suggest that the carboxylic acid group functions as the hydrogen-bond donor, the predicted p*K*_a_ values show unambigously that the OH groups are the proton donors, illustrating the significance of combining different information in the analysis of hydrogen bonds in proteins. The third group comprises hydrogen-bond interactions between OH groups and backbone amide O atoms. The interaction between Tyr30 and Glu202 is only observed in wild-type RGAE. In RGAE D192N Glu202 is involved in crystal packing, which explains why this hydrogen bond is not formed. The O⋯O distances vary between 2.62 (3) and 2.87 (2) Å, with the equivalent distances in the two structures being remarkably similar. In the small-molecule structures the average O⋯O distance is 2.77 (8) Å, with a fairly large spread (Fig. 5 ▶). The abundance and short distances made us look for a possible structural role for these inter­actions. Four interactions of this type connect residues in loops. The hydrogen bonds Thr86–Gly76, Ser44–Arg85 and Ser44–Arg85 connect different loops and Thr20–Gly17 (Fig. 6 ▶ *c*) forms a link between residues in the same loop. The three other hydrogen bonds between residues close in the sequence (Thr215–Ala211, Ser187–Thr184 and Thr49–Ala45) connect residues in the same α-­helix and may have a role in reducing the solvent-exposure of the helix.

The most likely candidate for the deshielded proton is that in the very short hydrogen bond between Asp75 and Asp87. This hydrogen bond is buried in the protein, with relative solvent accessibilities of 1% and 6% compared with Asp in an Ala-Asp-Ala sequence (Table 4 ▶). Asp75 is conserved in six of eight sequences in block III of conserved residues in the SGNH-family members analysed by Mølgaard *et al.* (2000 ▶) and is involved in a hydrogen-bond network to the oxyanion hole. The two carboxylic acid groups of Asp and Glu residues are normally predicted to have very similar p*K*_a_ values of around 4. Whereas Asp87 is not involved in any additional hydrogen bonds, Asp75 is also hydrogen bonded to a backbone amide proton (Fig. 3 ▶), which could explain why Asp75 titrates before Asp87.

#### 4.1.2. The N—H⋯O hydrogen bonds

An equivalent number of short N—H⋯O hydrogen bonds are observed in the RGAE structures. Five of these that are identical in the two structures are between backbone amide and amide O and define the secondary structure of the protein. Considering that the average N⋯O distance in small-molecule structures is 2.91 (7) Å (Fig. 5 ▶), it is interesting that these short interactions correspond to N—H⋯O hydrogen bonds that are buried or partly buried in the protein (Val3–Thr34 shown in Fig. 6 ▶ *d*). They contribute to the secondary structure of RGAE but not to a specific structural element. They are found in α-helices (intrahelical and interhelical) and β-sheets as well as in loops. The other N—H⋯O hydrogen bonds all have side chains as donors. The expected distance from the small-molecule structures for a His–Asp hydrogen bond is 2.75 (12) Å; the three inter­actions of this type found in wild-type RGAE are all shorter. Only one of these, His169–Glu70, is preserved in the RGAE D192N structure. Its environment (Fig. 6 ▶ *e*) is of the mixed polar/apolar character found for other buried His residues (Edgcomb & Murphy, 2002 ▶). This type of hydrogen bond, which is part of the catalytic machinery for proteases and esterases, has been discussed extensively owing to its importance in catalysis and as an LBHB. The hydrogen bonds involving the guanidinium group as donor are all longer than those involving His and are not likely to be candidates for a short hydrogen bond owing to the large difference in the p*K*_a_ values. The three inter­actions are all partly buried in the protein; the environment of the most buried Arg46-Asp82 is illustrated in Fig. 6 ▶(*f*).

#### 4.1.3. The low-field ^1^H NMR signals in RGAE

In the Biological Magnetic Resonance Bank (Seavey *et al.*, 1991 ▶; http://www.bmrb.wisc.edu), deshielded protons with chemical shifts around 18 p.p.m. are exclusively assigned to the hydrogen in His–Asp/Glu hydrogen bonds. Therefore, the short hydrogen bond between the active-site residues His195 and Asp192 [2.63 (2) Å] was initially thought to give rise to the low-field ^1^H NMR signal at approximately 18 p.p.m. (Mølgaard, 2000 ▶), as a similar hydrogen bond in the active site of α-chymotrypsin (Cassidy *et al.*, 1997 ▶) gave rise to a ^1^H NMR signal above 18 p.p.m. In other systems with analogous catalytic triads, signals at these p.p.m. values have been assigned to similar hydrogen bonds, which are often referred to as LBHBs. Their role and importance in enzymatic function have been debated for more than 10 y (see, for example, Schutz & Warshel, 2004 ▶; Frey *et al.*, 1994 ▶).

The calculated chemical shift value for the proton in the His195–Asp192 hydrogen bond was 18.1 p.p.m., but it is possible that the proton exchanges too fast with the solvent to be observed, since the residues have some solvent accessibility, as shown in Table 4 ▶. A ^1^H NMR pH profile from this active-site hydrogen bond (or any other with a His donor) would be most likely to give a titration curve (from the N^δ1^ proton) with a signal at approximately 18 p.p.m. from the doubly proton­ated His (at lower pH) and one at 14–15 p.p.m. (at higher pH) from the singly protonated His (Robillard & Shulman, 1972 ▶; Cassidy *et al.*, 1997 ▶).

In the spectra of the wild type (Fig. 4 ▶) a signal above 14 p.p.m. appears at higher pH values, but the 18.2 p.p.m. signal is present throughout the range. It is not unlikely that the signal around 18 p.p.m. contains a contribution from the His195 N^δ1^–Asp192 O^δ2^ hydrogen bond at low pH. Based on the structure of the D192N variant and the corresponding ^1^H NMR spectra we could rule out this active-site hydrogen bond as the sole origin of these signals, as the 18.2 p.p.m. signal persists in the spectra of RGAE D192N despite the mutation and the absence of the 14 p.p.m. signal in RGAE D192N.

The His169–Glu70 hydrogen bond with an N⋯O distance similar to the His195–Asp192 hydrogen bond is present in both wild-type RGAE and RGAE D192N and is located in the interior of the protein. The theoretical calculations for this interaction resulted in a chemical shift of 18.4 p.p.m. for the proton in the His169–Glu70 hydrogen bond. This value may well be overestimated since the corresponding optimized N⋯O distance of 2.55 Å is shorter than the experimental value of 2.61 Å. From the calculated p*K*_a_ values (Table 4 ▶), the 18 p.p.m. signal would only be observable at pH values below approximately 5, which is satisfied under the conditions of the crystal structures. There is sufficient space in the crystal structure to allow the conformational change that would be associated with the deprotonization of the charged imidazole system. At higher pH values the ^1^H NMR signal is expected to move to 14–15 p.p.m., giving rise to a titration curve as described above for the active-site (His195–Asp192) hydrogen bond. Since the His169–Glu70 hydrogen bond is present in both RGAE structures with identical distances at low pH and the 14 p.p.m. signal is only observed in the spectrum of wild-type RGAE, we would not expect the proton from the His169–Glu70 hydrogen bond to be responsible for the 18 p.p.m. signal at the higher pH values.

A hydrogen bond that differs between the wild type and the D192N variant is His193–Glu140 (Fig. 2 ▶). This bond is 2.66 Å in the wild type and approximately 0.2 Å longer in the structure of RGAE D192N. As a result of the differences in the structures it is an alternative candidate for the 14 p.p.m. signal in the spectra from wild-type RGAE. The calculated p*K*_a_ value of His193 (7.2) is consistent with the pH profile of the 14 p.p.m. signal. However, the side-chain accessibility is high, so that fast exchange with the solvent could cause problems in detecting the ^1^H NMR signal.

The Asp and Glu protons are not normally observed in protein NMR experiments, but from experiments on small molecules it is known that hydrogen bonds between two carboxylic acids (or carboxylic acid and carboxylate) can give rise to low-field ^1^H NMR signals (Brück *et al.*, 2000 ▶; Altman *et al.*, 1978 ▶; Jeffrey & Yeon, 1986 ▶) and that these are the shortest hydrogen bonds observed in organic molecules. In RGAE it is also this type of hydrogen bond that represents that with the shortest donor–acceptor distance.

The Asp75–Asp87 hydrogen bond is <2.5 Å in both structures. Asp75 is conserved in six of eight sequences in block III of the SGNH-family members analysed in Mølgaard *et al.* (2000 ▶) and is involved in a hydrogen-bond network close to the oxyanion hole. This hydrogen bond is buried in the protein and its hydrophobic environment would imply a calculated p*K*_a_ value of 10.2 for Asp87, corresponding to a hydrogen bond that is stable up to pH 10, where the protein starts to denature. The hydrophobic environment also prevents fast exchange with the solvent. The calculated chemical shift for the proton involved in the Asp75–Asp87 hydrogen bond is 18.5 p.p.m. with an associated O⋯O distance of 2.50 Å, which is in good agreement with the experimental value of 2.47 (2) Å. In the HIV protease system a similar short hydrogen bond between the catalytic Asps has been characterized by computational methods (Piana & Carloni, 2000 ▶; Porter & Molina, 2006 ▶) and shown to be an LBHB.

Taking all the evidence into account, we conclude that the Asp75–Asp87 hydrogen bond is the most likely origin of the 18.2 p.p.m. ^1^H NMR signal in both wild-type RGAE and RGAE D192N. This would be the first identification of a low-field ^1^H chemical shift for a short Asp–Asp hydrogen bond in a protein.

It is more difficult to assign the 14 p.p.m. ^1^H NMR signal that only occurs in the wild-type RGAE at pH > 8. The structural differences observed between RGAE and RGAE D192N are in the hydrogen-bonding system in the active site. This makes the two short hydrogen bonds found in wild-type RGAE, His195 N^δ1^–Asp192 O^δ2^ and His193 N^δ1^–Glu140 O^∊1^, the most likely candidates.

#### 4.1.4. Interactions between carboxylic acids in proteins

The very short buried hydrogen bond observed between Asp75 and Asp87 in RGAE triggered the question: how common are such short hydrogen bonds between carboxylic acids? An examination of a subset of protein chains with less than 30% sequence identity showed that in about 16% of these there were O⋯O distances between Asp and Asp, Asp and Glu or Glu and Glu that were smaller than 2.9 Å. A similar analysis of pairs of hydrogen-bonded carboxylic acid side chains has been carried out by Wohlfahrt (2005 ▶) based on 1600 chains from the 1999 release of the PDB (sequence identity < 90%, *R* values < 25% and resolution better than 2.5 Å). With the same distance cutoff, Wohlfahrt (2005 ▶) found short contacts between Asp and Glu residues in approximately 19% of the protein structures, which is slightly higher than our result. However, Wohlfahrt’s analysis was based on structures rather than protein chains and therefore also includes protein–protein interactions (crystal contacts, multimers *etc*.) which could be the origin of the difference. Our analysis also showed that in 9% of the structures examined the O⋯O distances were smaller than 2.6 Å, corresponding to very strong short hydrogen bonds. Very nice examples of hydrogen-bonded carboxy groups are found in the high-resolution structure of *Pseudomonas* serine-carboxyl proteinase (Wlodawer *et al.*, 2001 ▶).

In RGAE, the Asp–Asp hydrogen bond is found in close proximity of the oxyanion hole (Fig. 3 ▶) and at one point an Asp corresponding to Asp75 was, based on site-directed mutagenesis, thought to be a catalytic residue in a homologous lipase/acyltransferase from *Aeromonas hydrophila* (Brumlik & Buckley, 1996 ▶). Without specifying the exact role, this all indicates that the Asp75–Asp87 hydrogen bond in RGAE may be of importance for the function of this enzyme. In a study of the different catalytic units/motifs, Gutteridge & Thornton (2005 ▶) found that interactions between Asp and/or Glu (side-chain atoms closer than 4 Å) had one of the residues annotated as catalytic more often than expected. Along with our results, this indicates that the short hydrogen bonds between carboxylic acid residues could exert a variety of roles in the catalytic function of enzymes. It might even be possible to use these hydrogen bonds as pointers towards functionally important areas in enzymes with unknown activity.

## 5. Conclusions

Despite the precedents in the literatue, our results show that the low-field signals observed in some ^1^H NMR experiments on proteins cannot be ascribed to active-site hydrogen bonds without additional evidence. The 18 p.p.m. ^1^H NMR signal in RGAE cannot be assigned to the hydrogen bond between the residues in the catalytic triad. The protein contains other hydrogen bonds that are so short that the proton signal is shifted to the 18 p.p.m. region. This was supported by calculations of predicted chemical shifts for a few specific hydrogen bonds. Based on the ^1^H NMR spectra, analysis of the X-­ray structures and computational methods, we concluded that the proton in the Asp75–Asp87 hydrogen bond contributes to the 18 p.p.m. ^1^H NMR signal observed for both wild-type and D192N RGAE. Our analysis of the short hydrogen bonds in RGAE revealed that the short hydrogen bonds are located close to the active site, indicating a role in the enzymatic function. Interactions between carboxylic acid side chains are not rare, as our search in a PDB subset revealed short contacts between carboxylic acid side chains in 16% of the protein chains. Many of the shortest contacts involve residues that are putative catalytic residues or residues close to the active site, which emphasizes the importance of including Asp and Glu as possible hydrogen-bond donors.

## Supplementary Material

PDB reference: D192N rhamnogalacturonan acetylesterase, 3c1u, r3c1usf

## Figures and Tables

**Table 1:** Data-collection and refinement statistics for RGAE D192N Values in parentheses are for the outer resolution shell.

Total No. of reflections	497682
Unique reflections	55818
Resolution range (Å)	19.73–1.33 (1.35–1.33)
Completeness (%)	99.5 (98.3)
*I*/σ(*I*) > 2 (%)	91.3 (72.8)
*R*_merge_ (%)	3.2 (19.8)
Average redundancy	8.9 (2.3)
*R*, all data† (%)	11.57
*R*, *F*_o_ > 4σ(*F*_o_)† (%)	10.98
*R*_free_, all data‡ (%)	15.34
*R*_free_, *F*_o_ > 4σ(*F*_o_)‡ (%)	14.56
No. of non-H atoms in model§	
Protein	1768
GlcNAc + acetate	32
Water molecules	355
〈*B*〉§ (Å^2^)	
Protein main chain	13.3
Protein side chain	15.6
GlcNAc + acetate	35.6
Water molecules	37.3
Ramachandran statistics¶	
Core (%)	99.0
Outliers (%)	1.0

† Using all reflections (work and free set).

‡ Based on 5% randomly chosen reflections.

§ Including disordered atoms.

¶ Kleywegt & Jones (1996 ▶). The *STructure ANalysis* (*STAN*) server (http://xray.bmc.uu.se/cgi-bin/gerard/rama_server.pl) was used.

**Table 2:** Short hydrogen bonds with O—H donors from the Cambridge Structural Database Mean distances and angles are given along with the sample standard deviation†. *D*, donor; *A*, acceptor.

Donor group	Acceptor group	No. of fragments	*d*(*D*⋯*A*) (Å)	∠(*D*—H⋯*A*) (°)
Carboxylic acid	Carboxylate	337	2.54 (6)	169 (8)
Carboxylic acid	Amide O	101	2.60 (5)	165 (9)
Phenol	Carboxylate	44	2.64 (8)	169 (8)
Carboxylic acid	Aliphatic OH	199	2.65 (5)	164 (9)
Carboxylic acid	Carboxylic acid	1358	2.65 (4)	171 (7)
Carboxylic acid	Phenol	18	2.67 (7)	166 (10)
Phenol	Amide O	19	2.70 (7)	168 (9)
Phenol	Aliphatic OH	137	2.73 (8)	162 (11)
Phenol	Carboxylic acid	35	2.74 (10)	160 (15)
Aliphatic OH	Carboxylate	221	2.74 (10)	162 (12)
Aliphatic OH	Amide O	65	2.77 (8)	164 (10)
Aliphatic OH	Aliphatic OH	4931	2.78 (8)	163 (11)
Phenol	Phenol	514	2.80 (9)	159 (14)
Aliphatic OH	Imidazole	7	2.80 (14)	162 (12)
Aliphatic OH	Carboxylic acid	232	2.81 (9)	157 (14)
Aliphatic OH	Phenol	93	2.82 (9)	162 (13)

† Sample standard deviation = 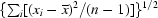.

**Table 3:** Short hydrogen bonds with N—H donors from the Cambridge Structural Database Mean distances and angles are given along with the sample standard deviation†. *D*, donor; *A*, acceptor.

Donor group	Acceptor group	No. of fragments	*d*(D⋯*A*) (Å)	∠(*D*—H⋯*A*) (°)
Imidazole	Carboxylate	53	2.75 (12)	164 (12)
Imidazole	Aliphatic OH	10	2.81 (5)	166 (10)
Aliphatic NH^+^	Carboxylate	306	2.82 (8)	161 (12)
Imidazole	Carboxylic acid	9	2.83 (9)	155 (23)
Indole	Carboxylate	8	2.84 (7)	161 (10)
Imidazole	Amide O	5	2.85 (13)	162 (4)
Aliphatic NH^+^	Amide O	16	2.85 (7)	151 (18)
Aliphatic NH^+^	Aliphatic OH	24	2.85 (9)	157 (14)
Indole	Aliphatic OH	15	2.87 (5)	159 (13)
Imidazole	Imidazole	63	2.88 (7)	167 (8)
Backbone NH	Carboxylate	43	2.89 (6)	160 (11)
Indole	Amide O	11	2.89 (6)	162 (11)
Aliphatic NH^+^	Carboxylic acid	52	2.89 (8)	149 (15)
Aliphatic NH^+^	Phenol	15	2.89 (7)	153 (13)
Guanidinium	Carboxylate	59	2.90 (7)	163 (12)
Backbone NH	Amide O	415	2.91 (7)	162 (11)
Backbone NH	Aliphatic OH	22	2.93 (5)	164 (13)
Guanidinium	Aliphatic OH	5	2.93 (5)	161 (7)
Amide NH side chain	Amide O	65	2.93 (6)	165 (10)
Indole	Carboxylic acid	22	2.93 (8)	159 (11)
Guanidinium	Carboxylic acid	12	2.93 (7)	161 (13)
Backbone NH	Carboxylic acid	61	2.95 (6)	161 (10)
Amide NH side chain	Carboxylate	15	2.95 (5)	159 (15)
Guanidinium	Phenol	5	2.96 (6)	150 (10)
Backbone NH	Phenol	7	2.97 (7)	160 (13)
Amide NH side chain	Aliphatic OH	23	2.97 (7)	160 (13)
Amide NH side chain	Carboxylic acid	5	2.98 (4)	165 (6)

† Sample standard deviation = 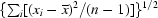.

**Table 4:** Short hydrogen bonds in wild-type RGAE and RGAE D192N The estimated standard deviations on the distances were obtained from matrix inversion in *SHELXL* least-squares refinements. All ∠C—N⋯O, ∠C—O⋯N and ∠C—O⋯O are larger than 90°. The distances in square parentheses are included for comparison, but are not considered to be potential hydrogen bonds. The interactions highlighted in bold are illustrated in Figs. 2 ▶, 3 ▶ and 6 ▶.

Donor–acceptor (or *vice versa*)	Distance in wild type (Å)	Distance in D192N (Å)	Relative accessibility† (%)	p*K*_a_‡
**Asp75 O^δ2^—Asp87 O^δ2^**	2.47 (1)	2.48 (2)	1/6	4.1/10.2
Glu202 O^∊2^—Glu206 O^∊2^	2.50 (2)	[5.48 (4)]	27/25	2.6/8.7
Tyr30 O^H^—Glu202 O^∊2^	2.63 (1)	[6.50 (2)]	21/27	12.3/2.6
Thr86 O^γ1^—Gly76 O	2.72 (1)	2.62 (3)	5/27	—/—
Ser187 O^γ^—Thr184 O	2.69 (2)	2.65 (2)	64/38	—/—
Ser44 O^γ^—Arg85 O	2.67 (1)	2.69 (2)	0/37	—/—
**Thr20 O^γ1^—Gly17 O**	2.69 (2)	2.71 (3)	5/39	—/—
Thr49 O^γ1^—Ala45 O	2.77 (1)	2.73 (2)	39/0	—/—
Thr194 O^γ1^—Asn136 O^δ1^	2.87 (2)	2.75 (2)	18/38	—/—
Ser218 O^γ^—Glu165 O^∊1^	2.56 (2)	2.56 (3)	30/33	—/3.3
**Thr10 O^γ1^—Asp8 O^δ1^**	2.67 (1)	2.62 (2)	8/2	—/4.6
**Ser98 O^γ^—Asp82 O^δ2^**	2.64 (2)	2.63 (3)	15/20	—/0.3
Ser171 O^γ^—Asp168 O^δ2^	2.66 (1)	2.69 (3)	29/26	—/2.4
Ser131 O^γ^—Glu70 O^∊1^	2.75 (1)	2.74 (2)	1/4	—/3.6
**His195 N^δ1^—Asp192 O^δ2^**	2.63 (2)	—	20/40	5.1/1.7
**His195 N^δ1^—Asn192 O^δ1^**	—	[4.38 (2)]§	10[Table-fn tfn10]/49[Table-fn tfn10]	2.9[Table-fn tfn10]/—
**His169 N^δ1^—Glu70 O^∊1^**	2.61 (1)	2.62 (2)	0/3	4.6/3.6
His193 N^δ1^—Glu140 O^∊1^	2.66 (4)	2.88 (4)	41/60	7.2/4.0
Arg53 N^∊^—Glu51 O^∊1^	2.78 (2)	2.75 (2)	30/41	11.8/3.5
**Arg46 N^H1^—Asp82 O^δ1^**	2.76 (2)	2.80 (3)	0/20	11.6/0.3
Arg43 N^∊^—Glu51 O^∊1^	2.80 (2)	2.78 (2)	26/41	11.6/3.5
**Val3 N—Thr34 O**	2.77 (1)	2.75 (2)	0/61	—/—
Glu115 N—Pro111 O	2.79 (1)	2.78 (2)	14/0	—/—
Arg85 N—Asn83 O^δ1^	2.80 (2)	2.80 (3)	37/55	—/—
Ser197 N—Tyr188 O	2.83 (2)	2.79 (2)	15/9	—/—
Ala201 N—Ser197 O	2.84 (1)	2.79 (2)	0/15	—/—
His195 N^δ1^—Ser9 O^γ^	—	2.73 (2)	10[Table-fn tfn10]/0[Table-fn tfn10]	2.9[Table-fn tfn10]/—
His195 N^∊2^—Ser9 O^γ^	[2.95 (2)]	—	20/7	5.1/—
Lys124 N^ζ^—Ala58 O	2.66 (2)	[4.64 (6)]	43/12	9.8/—
His169 N^∊2^—Val204 O	2.79 (1)	2.73 (2)	41/3	7.2/—

† Relative residue accessibility in wild-type RGAE compared with Ala-*X*-Ala, using a default probe size of 1.4 Å.

‡ Listed p*K*_a_ values are based on the wild-type structure.

§ Distance with side-chain configuration as in Fig. 2 ▶. The shortest distance between any two atoms in the two side chains is 3.36 Å.

¶ Accessibility/p*K*_a_ calculated for the RGAE D192N structure.
